# Predicting adherence in routine internet based cognitive behavioural therapy for depression: Retrospective cohort study

**DOI:** 10.1192/j.eurpsy.2023.1812

**Published:** 2023-07-19

**Authors:** E. K. Jensen, K. Mathiasen, H. Riper, M. B. Lichtenstein

**Affiliations:** 1 Centre for Digital Psychiatry, Mental Health Services Region of Southern Denmark, Odense C; 2 Department of Clinical Research, University of Southern Denmark, Odense M, Denmark; 3 Faculty of Behavioural and Movement Sciences, Clinical Psychology, Vrije Universiteit, Amsterdam, Netherlands

## Abstract

**Introduction:**

On average, thirty percent of patients in internet based treatments do not complete the treatment program. The majority of studies predicting adherence have focused on baseline variables. While some consistent predictors have emerged (e.g. gender, education), they are insufficient for guiding clinicians in identifying patients at risk for dropout. More precise predictors are needed. More recently, studies on prediction have started to explore process variables such as early response to treatment or program usage.

**Objectives:**

To investigate:

- How much variance in adherence is explained by baseline symptoms and sociodemographic variables?
- Can we improve the model by including early response and program usage as predictors?
- What is the predictive accuracy of the most parsimonious regression model?

**Methods:**

Data will be extracted from the Danish ‘Internetpsychiatry’ clinic, which delivers guided internet based cognitive behavioural therapy for depression. Sociodemographic data is collected upon application, and symptoms of depression and anxiety are measured at the start of treatment. Further, symptoms of depression are measured between each session of the online treatment program. Early response to treatment will be conceptualized as the individual regression slope of depression scores for each patient, during the first four weeks of treatment. Program usage data will be collected from the online treatment platform (e.g. number of words per message to therapists, time spent on each session during the first four weeks, number of logins during the first four weeks).

Predictors for adherence will be examined in a hierarchical logistic regression. Models will be compared using ANOVA. The most parsimonious model will be determined using the Aikake Information Criterion. Receiver operating characteristic curve analyses will be used to classify the accuracy of the model.

**Results:**

Analyses have not yet been conducted. Results will be available for presentation at the conference.

**Conclusions:**

Determining more accurate predictors for adherence in internet based treatments is the first step towards improving adherence. Research findings need to be translated into clinically useful guidelines that may inform clinical decision making. Findings from this study could potentially be implemented as a system that monitors patients’ program usage and symptom development and signals therapists if a patient is at risk for dropout.

**Disclosure of Interest:**

None Declared

